# Identifying characteristics of patients requiring proactive pharmaceutical interventions in the recovery period and assessing the effect of rehabilitation and drugs: a retrospective study

**DOI:** 10.1186/s40780-025-00435-4

**Published:** 2025-04-08

**Authors:** Megumi Yahara-Hotta, Tomoyuki Ogino, Kisako Higa, Moka Yamakawa, Toshiyuki Shikata, Yoshihiro Kanata, Kenji Ikeda, Atsushi Kinoshita

**Affiliations:** 1 School of Pharmacy, Hyogo Medical University, 1-3-6 Minatojima, Chuo-Ku, Kobe, Japan; 2 Clinical Pharmacy Research and Education Unit, Graduate School of Pharmaceutical Sciences, Osaka University, 1-6 Yamadaoka, Suita, Osaka Japan; 3 School of Rehabilitation, Hyogo Medical University, 1-3-6 Minatojima, Chuo-Ku, Kobe, Japan; 4 Department of Pharmacy, Hyogo Medical University Sasayama Medical Center, 5 Kuroka, Tambasasayama, Hyogo Japan; 5 Department of Rehabilitation, Hyogo Medical University Sasayama Medical Center, 5 Kuroka, Tambasasayama, Hyogo Japan

**Keywords:** Rehabilitation, Medication, Older, Pharmaceutical, Functional independence measure, Recovery period, Activities of daily living

## Abstract

**Background:**

The aging of the population in many countries has made rehabilitation an essential part of improving the quality of life of older individuals. The risk factors for falls during rehabilitation include a history of falls, gait disturbances, dizziness, and medication use. Although numerous studies have explored various fall prevention measures, stratified or detailed analyses of the relationship between the activities of daily living (ADL) and drugs have not been performed. This study aimed to examine the effect of drugs on ADLs in patients undergoing rehabilitation and explored the factors affecting patients’ ADLs identify the characteristics of patients requiring proactive pharmaceutical interventions.

**Methods:**

Participants aged ≥ 20 years admitted to the Kaifukuki Rehabilitation Ward at Hyogo Medical University Sasayama Medical Center underwent functional independence measure (FIM) assessments and were evaluated for medication use. The complexity of the medication regimen was assessed using the Japanese version of the medication regimen complexity index (MRCI-J) based on prescription data. Hierarchical cluster analysis was used to classify the participants based on their FIM scores.

**Results:**

No correlation was found between FIM motor gain and MRCI-J differences among all participants. Hierarchical cluster analysis was used to classify participants into four groups based on their FIM motor and cognitive scores at admission and discharge. Decision tree analysis was performed using the four identified groups as objective variables and yielded eight nodes. The algorithm included length of hospital stay, sex, age, units of rehabilitation performed, and the MRCI-J score. The group with a hospital stay < 74 days, aged < 90 years, and who underwent > 77 units of rehabilitation during the study period was further divided into fourth tiers based on the MRCI-J scores, with the non-increased MRCI-J group assigned as Node 7 and the increased MRCI-J group as Node 8.

**Conclusions:**

No relationship was found between ADLs and prescribed drugs in the overall participant population. In participants from Nodes 7 and 8, who had a relatively short length of hospital stay and were discharged with preserved physical and cognitive functions, prescription changes appeared to have some effects on patient’s ADLs.

**Supplementary Information:**

The online version contains supplementary material available at 10.1186/s40780-025-00435-4.

## Background

The aging of the population in many countries has made rehabilitation an essential part of improving the quality of life of older individuals. Older individuals may require long-term care owing to changes in their physical and cognitive functions. In Canada, public health insurance coverage for older care is limited and does not include home care, which is primarily provided by informal caregivers [1]. In the Kingdom of Sweden, the Social Services Act mandates the provision of care services for older adults, including institutional services [2]. In Japan, the aging of the population has led to an increase in the number of caregivers, and the long-term care insurance system has been established as a societal mechanism to support long-term care.

“Falls and fractures”are significant factors that lead to the need for nursing care. Kanamaru et al. indicated that proximal femur fracture is a poor prognostic factor for life expectancy, suggesting that early postoperative discharge can prevent the occurrence of new complications and reduce mortality [3]. Early rehabilitation is essential for patients with proximal femur fractures to facilitate early discharge. However, rehabilitation also carries the risk of causing another fall, requiring careful intervention. Deandrea et al. reported that a history of falls, gait disturbances, dizziness, and medication use are risk factors for falls [4]. One approach to reducing these risks is by implementing measures to prevent drug-induced dizziness and lightheadedness. Kishimoto et al. suggested that certain medications could interfere with rehabilitation and lead to a decline in the activities of daily living (ADLs). Hence, pharmacists should intervene to individually optimize drug therapy for patients in recovery, recommending the most suitable medications [5]. Despite the existence of many fall prevention measures for the older population, a study on pharmacist intervention for patients taking medications known to increase the risk of falls found no significant difference in the recurrence or incidence of falls over a 1-year period [6]. Although patient education provided by general practitioners resulted in a decrease in the incidence of falls, the impact of the intervention was not significant [7]. However, most previous studies have only analyzed participants as a whole, with no stratified, in-depth analyses of the factors influencing fall risk. Additionally, all patients in the Kaifukuki Rehabilitation Wards included in this study receive some form of rehabilitation, with their functional independence measure (FIM) scores regularly monitored for improvement throughout their treatment. As a result, FIM scores generally tend to improve. Given this, there is a possibility that certain medications may become unnecessary, or their dosages may be reduced. However, the interaction between rehabilitation and medication use remains unclear.

This study aimed to examine the influence of drugs on ADLs of patients undergoing rehabilitation, a retrospective study of patient backgrounds, ADL modifications, and changes in prescription medications, and to identify the relationship between FIM score change and the medication regimen complexity index (MRCI-J) scores in patients in Kaifukuki Rehabilitation Wards.

## Methods

This retrospective observational study was conducted at the Hyogo Medical University (HMU) Sasayama Medical Center. This core hospital is dedicated to supporting the health of residents and has a treatment system tailored to meet regional needs. The hospital has 180 beds, of which 44 are in the Kaifukuki Rehabilitation Ward. Unlike the “convalescent rehabilitation wards”in other countries, this facility is specifically referred to as the “Kaifukuki Rehabilitation Ward”in Japan [8].

### Participants

The study included individuals aged ≥ 20 years; admitted to the Kaifukuki Rehabilitation Ward at the HMU Sasayama Medical Center on or after April 1, 2022; and discharged on or before March 31, 2023. Patients who underwent rehabilitation performed by a physical, occupational, or speech-language-hearing therapist; underwent at least two FIM [9] evaluations during consecutive hospitalizations; had a history of drug use, excluding injection prescriptions, at admission and discharge; and did not provide informed consent for the use of their medical records were excluded.

### Data collection and processing

First, participants who met the eligibility criteria were identified using an electronic medical record system that manages patients who have undergone rehabilitation. The length of hospital stay was determined based on the admission and discharge dates, whereas the total number of rehabilitation units stay, sex, age, and primary diagnosis were extracted from the electronic medical record system. One unit of rehabilitation was defined as 20 min of rehabilitation [10]. The FIM scores were also obtained from the electronic medical records. Diseases were classified using the International Classification of Diseases, 11th version (ICD-11) [11]. Additionally, the prescription drug information at admission and discharge were extracted from the electronic medical record system. The MRCI-J scores were calculated using these data to determine the changes in prescription complexity.

### Functional independence measure

The participants’ ADLs were assessed at admission and discharge using the FIM [9]. The FIM is an 18-item, 7-level scale (with scores ranging from 1–7) developed to evaluate the severity of disability and medical rehabilitation functional outcomes. It has two major components: 13 motor items assessing self-care, defecation control, transfers, and mobility, and 5 cognitive items evaluating communication and social cognition. In this study, the FIM motor scores were categorized as follows: < 50 points as requiring complete assistance, 50–70 points as requiring semi-assistance, and ≥ 70 points as indicating independent self-care [12]. For the FIM cognitive items, a cutoff score of 20 [12, 13] was used, with a score < 20 indicating low cognitive function.

### Measure of the medication regimen complexity index

The MRCI-J [14] was used to assess the complexity of participants’ prescription regimens based on prescription drug information. The MRCI-J consists of three sections that account for various aspects of drug therapy complexity. Section A assigns weights according to the route of administration (oral, topical, otic, or ophthalmic) and dosage form (tablet, liquid, or spray). Section B assigns weights according to the dosing frequency and daily timing. Section C assigned weights based on additional instructions required for administration (tapering schedules, food-related requirements, and crushing instructions). The MRCI-J checklist comprises 61 items, with higher total scores reflecting greater prescription complexity.

### Primary and secondary endpoints

The primary endpoint was the evaluation of the relationship between FIM score change and MRCI-J score. The secondary endpoints included stratification according to the participants’ backgrounds, examining the relationship between MRCI-J and length of hospital stay, rehabilitation units, age, and sex, and determining the trends in disease classifications.

### Definitions

In this study, MRCI-J scores that remained unchanged or improved were classified as non-increased MRCI-J, whereas worsening MRCI-J scores were categorized as increased MRCI-J. The FIM motor gain was calculated as the FIM motor score at discharge minus the FIM motor score at admission.

### Sample size calculation

There was no evidence on the association between the FIM motor gain and difference in MRCI-J scores. The cutoff value of the optimal MRCI-J score for changes in FIM motor gain is also unknown. Thus, it was not possible to estimate the sample size required for this study could not be calculated [15]. In addition, the small sample size of this pilot study, no inferential statistics were conducted [16].

### Statistical analysis

The following analysis methods were used for the purpose. A *p*-value < 0.05 was considered significant. All statistical analyses were performed using the JMP Pro® 16 software (SAS Institute, Cary, NC, USA).

- To examine whether medications had an effect on the patients’ ADLs, Spearman’s correlation coefficient was calculated using the difference between the FIM motor gain and changes in MRCI-J scores as continuous variables.
-  In order to understand the trends and characteristics of ADL changes in recovery patients, stratification was conducted by performing a hierarchical cluster analysis [17] using the FIM motor and cognitive items at admission and discharge serving as explanatory variables. The FIM score was treated as a continuous variable. Clustering is a method that calculates the similarity (distance) between samples based on the characteristics of the data, and groups data that are close to each other. Clustering was performed by three analysts using the Ward method, with the Euclidean square distance calculated as the sum of squares between each question item. The number of clusters were determined based on the dendrogram and distance graphs as sufficiently explaining the characteristics of the clusters. At this stage, using the previously reported ADL classification as a reference [12], the characteristics of each cluster obtained from the hierarchical cluster analysis were defined to establish a reasonable hierarchical structure.
- To search for the variables related to the characteristics of each cluster, a decision tree analysis was subsequently performed using the calculated clusters as objective variables and sex, age, length of hospital stay, number of rehabilitation units, and MRCI-J scores as explanatory variables were identified during the analysis. Sex, age, and MRCI-J scores were considered categorical variables. Decision tree analysis is a method of finding explanatory variables that influence the objective variable using a tree diagram called a decision tree. The likelihood ratio chi-square statistics were used as the criteria for variable selection to deal with the small samples and categorical data. The validity of the split was determined from the chi-square statistics and *p*-value.
- To further explore the characteristics of each node, Spearman’s correlation coefficients were calculated for the following variables: age, length of hospital stay, FIM motor gain, FIM cognitive items at admission and discharge, total FIM scores at admission and discharge, and MRCI-J scores at admission and discharge. The number of rehabilitation units was divided according to the length of hospital stay to obtain the units of rehabilitation per day. Spearman’s correlation test was used to determine the correlations, with a magnitude of 0.50–1.00 (− 1.00 to − 0.50) indicating a strong correlation [18].
- Partial correlation coefficients were calculated to account for potential confounders.
- Unpaired t-tests were carried out to compare the prescription complexity factors between nodes.

## Results

### Participants’ characteristics

In this study, 110 patients who were admitted to and discharged within the eligible period and had a history of admission to the Kaifukuki Rehabilitation Ward were selected. Two patients who lacked data on drug use upon admission and discharge were excluded. Finally, only 108 patients who met the eligibility criteria were analyzed; their backgrounds are presented in Table 1. The participants had a male to female ratio of 43:65, and their diseases were categorized according to the ICD-11 criteria [11]. The top three disease groups were injury, poisoning, and other consequences of external causes (59 cases); nervous system diseases (33 cases); and musculoskeletal system or connective tissue diseases (25 cases). The mean (standard deviation [SD]) values for each item were 78.9 (11.9) for age, 61.0 (38.5) days for the length of hospital stay, 148.3 (124.3) for the number of rehabilitation units, 74.1 (27.1) for the total FIM score at admission, 100.6 (26.6) for the total FIM score at discharge, 46.4 (21.3) for the total FIM motor score at admission, 71.1 (21.1) for the total FIM motor score at discharge, 27.7 (7.9) for the total FIM cognitive score at admission, 29.6 (6.8) for the total FIM cognitive score at discharge, 20.8 (13.5) for the MRCI-J score at admission, and 16.4 (11.4) for the MRCI-J score at discharge.

**Table 1:** Participants’ characteristics

Survey item	Total number of participants (*n* = 108)	
Female sex, *n* (%)	65 (60.2)	
International Classification of Diseases 11th Revision (ICD-11) codes, cases	Injury, poisoning or certain other consequences of external causes	59
	Diseases of the nervous system	33
	Diseases of the musculoskeletal system or connective tissue	25
	Diseases of the circulatory system	5
	Diseases of the respiratory system	5
	Certain infectious or parasitic diseases	2
	Diseases of the genitourinary system	2
	Neoplasms	1
	Diseases of the immune system	1
	Median (range)	Mean (SD)
Age, years	82.0 (43–99)	78.9 (11.9)
Length of stay in hospital, days	56 (8–187)	61.0 (38.5)
Units of rehabilitation during the period (1unit = 20 min)	115.0 (11–693)	148.3 (124.3)
Total FIM score at admission	76.0 (18–123)	74.1 (27.1)
Total FIM score at discharge	110.5 (20–126)	100.6 (26.6)
Total FIM motor score at admission	44.5 (13–91)	46.4 (21.3)
Total FIM motor score at discharge	80.0 (13–91)	71.1 (21.1)
Total FIM cognitive score at admission	29.0 (5–35)	27.7 (7.9)
Total FIM cognitive score at discharge	32.0 (7–35)	29.6 (6.8)
Total MRCI-J score at admission	19.0 (2.0–64.0)	20.8 (13.5)
Total MRCI-J score at discharge	14.0 (2.0–67.5)	16.4 (11.4)

*Abbreviations*: *FIM* Functional independence measure, *MRCI-J* the Japanese version of the medication regimen complexity index

### Causal relationship between the FIM motor gain and difference in MRCI-J scores

To assess the effect of ADLs and prescribed medications, the correlation between the FIM motor gains and difference in MRCI-J scores was examined for all participants. The correlation coefficient was − 0.07 (*p* = 0.47).

### Stratification based on FIM score

**Fig. 1:**
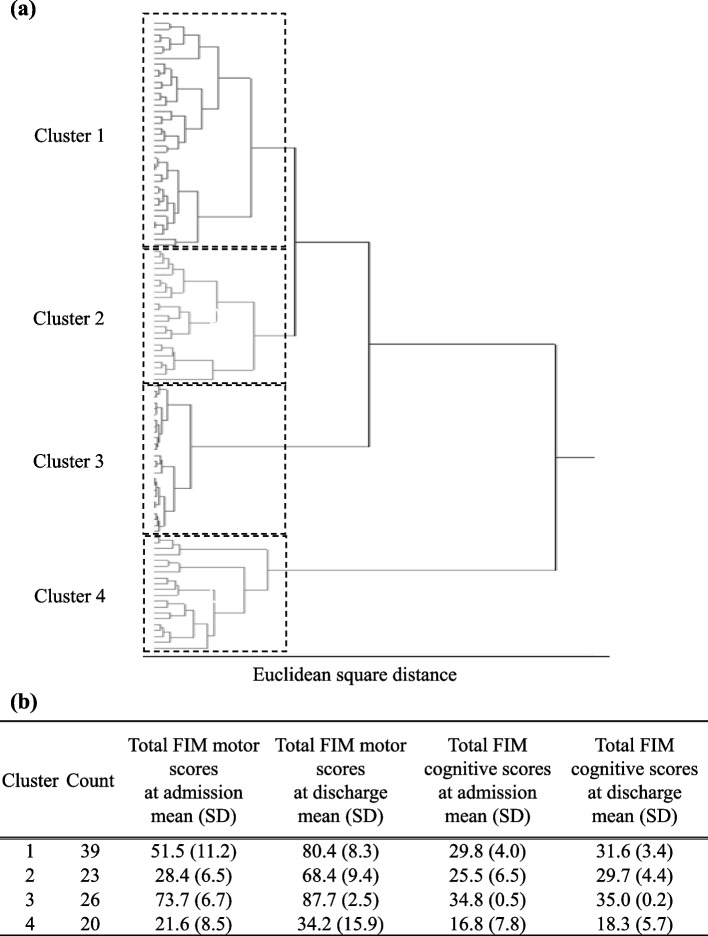
Hierarchical cluster analysis of participants based on FIM scores. **a **This is the Dendrogram. **b **The mean of each cluster

The participants were classified into four groups using hierarchical cluster analysis based on their FIM motor and cognitive scores (Fig. 1). Cluster 1 consisted of 39 participants, with mean (SD) FIM motor scores of 51.5 (11.2) at admission and 80.4 (8.3) at discharge and mean (SD) FIM cognitive scores of 29.8 (4.0) at admission and 31.6 (3.4) at discharge. Cluster 2 consisted of 23 participants, with mean (SD) FIM motor scores of 28.4 (6.5) at admission and 68.4 (9.4) at discharge and mean (SD) FIM cognitive scores of 25.5 (6.5) at admission and 29.7 (4.4) at discharge. Cluster 3 consisted of 26 participants, with mean (SD) FIM motor scores of 73.7 (6.7) at admission and 87.7 (2.5) at discharge and mean (SD) FIM cognitive scores of 34.8 (0.5) at admission and 35.0 (0.2) at discharge. Cluster 4 consisted of 20 participants, with mean (SD) FIM motor scores of 21.6 (8.5) at admission and 34.2 (15.9) at discharge and mean (SD) FIM cognitive scores of 16.8 (7.8) at admission and 18.3 (5.7) at discharge.

### Examination of factors affecting the ADL

**Fig. 2:**
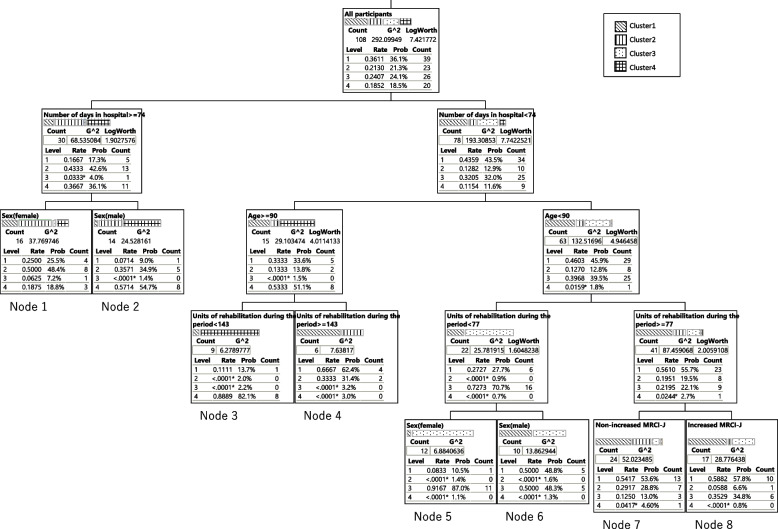
Decision tree analysis for examining factors affecting FIM scores. Supplementary Spearman’s correlation coefficient for each node. ** < 0.01, * < 0.05

The results of the decision tree analysis using the four clusters obtained from the hierarchical cluster analysis as objective variables are presented in Fig. 2, whereas the participants’ backgrounds for each node are listed in Table 2. In this model, the length of hospital stay was the primary determinant in the first tier; the participants were divided into two groups based on the length of hospital stay. In the second tier, participants with a length of hospital stay ≥ 74 days (28% of the total) were further divided according to sex, with Node 1 comprising the female group and Node 2 comprising the male group. Meanwhile, those with a length of hospital stay < 74 days (72% of the total) were further divided based on age (≥ 90 and < 90 years). In the third tier, participants aged ≥ 90 years (14% of the total) were further divided based on the number of rehabilitation units performed during the study period, with the group with fewer than 143 units designated as Node 3 and that with ≥ 143 units designated as Node 4. The group aged < 90 years (58% of the total) was grouped based on the number of rehabilitation units performed during the third tier. In the fourth tier, the group with < 77 units of rehabilitation (20% of the total) was further divided according to sex, with Node 5 comprising the female group and Node 6 comprising the male group. In the same tier, participants who underwent ≥ 77 units of rehabilitation during the study period (38% of the total) were further divided according to their MRCI-J score status. The group whose MRCI-J score remained unchanged was designated as Node 7, while that whose MRCI-J score increased was designated as Node 8. The FIM motor gains influenced the increase in the MRCI-J score, with Node 7 showing a significantly higher mean FIM motor gain score (standard error) compared with Node 8 (Node 7 vs. Node 8 = 31.1 (2.6) vs. 22.1 (3.1), unpaired t-test *p* < 0.05).

**Table 2:** Participants’ backgrounds for each node

Items	Node 1 (*n* = 16)		Node 2 (*n* = 14)		Node 3 (*n* = 9)		Node4 (*n* = 6)		Node 5 (*n* = 12)		Node 6 (*n* = 10)		Node 7 (*n* = 24)		Node 8 (*n* = 17)	
	Mean	SD	Mean	SD	Mean	SD	Mean	SD	Mean	SD	Mean	SD	Mean	SD	Mean	SD
Age, years	84.3	12.4	72.0	9.1	94.4	3.4	93.0	2.4	72.5	9.0	73.0	11.5	76.1	11.3	78.5	8.2
Units of rehabilitation per day	2.2	0.8	2.6	1.5	1.7	0.9	3.1	0.6	2.3	0.7	2.1	0.8	2.7	0.5	2.7	0.8
Total FIM score at admission	58.9	21.1	42.9	18.7	48.4	17.7	64.0	8.1	106.0	9.2	100.4	13.9	75.5	18.4	90.9	19.6
Total FIM score at discharge	92.5	24.7	72.6	31.9	64.3	23.7	97.2	10.6	122.0	3.6	120.0	6.8	108.2	18.5	114.5	10.4
FIM motor gain	32.3	14.1	24.8	21.4	14.1	8.7	29.5	8.9	15.9	6.8	18.6	15.1	31.1	12.5	22.1	13.5
Total FIM cognitive score at admission	25.6	8.7	19.9	9.6	19.6	8.5	25.8	3.0	34.4	1.7	32.7	4.1	29.6	5.0	30.6	5.1
Total FIM cognitive score at discharge	26.9	8.7	24.7	8.2	21.3	7.0	29.5	2.3	34.5	1.7	33.7	3.1	31.2	4.1	32.2	4.2
Total MRCI-J score at admission	23.6	10.5	28.5	14.7	16.4	9.9	15.5	6.7	18.3	15.5	15.1	8.7	24.7	12.7	15.9	16.9
Total MRCI-J score at discharge	16.5	6.4	22.5	12.1	11.2	7.1	10.9	2.8	14.5	14.9	10.7	6.1	17.0	7.9	19.8	18.0

*Abbreviations*: *FIM* Functional independence measure, *MRCI-J* the Japanese version of the medication regimen complexity index

### Examining the relationship between the prescribed medications and other variables at each node

To determine the relationship between rehabilitation and prescribed medications per node, the Spearman’s correlation coefficients are presented in Supplementary. The following data outline the correlations within the range of 0.50 − 1.00 (− 1.00 to − 0.50), defined as strong in this study, and demonstrated significant correlations between certain factors and the MRCI-J score. Node 6 showed significant correlations between the FIM cognitive score at admission and MRCI-J score at discharge (rs = − 0.7, *p* < 0.05). Node 8 showed significant correlations between the FIM motor gain and MRCI-J score at admission (rs = − 0.5, *p* < 0.05), FIM motor gain and MRCI-J at discharge (rs = − 0.6, *p* < 0.05), FIM cognitive score at admission and MRCI-J score at admission (rs = 0.6, *p* < 0.05), FIM cognitive score at discharge and MRCI-J score at admission (rs = 0.7, *p* < 0.01), FIM cognitive score at discharge and MRCI-J score at discharge (rs = 0.6, *p* < 0.05), total FIM score at admission and MRCI-J score at admission (rs = 0.6, *p* < 0.01), total FIM score at admission and MRCI-J score at discharge (rs = 0.6, *p* < 0.01), total FIM score at discharge and MRCI-J score at admission (rs = 0.5, *p* < 0.05), and total FIM score at discharge and MRCI-J score at discharge (rs = 0.5, *p* < 0.05). This should include the findings of the study including, if appropriate, results of statistical analysis which must be included either in the text or as tables and figures. However, a partial correlation coefficient was calculated to remove the influence of all other variables, and no difference was found.

## Discussion

Some patients undergoing convalescent rehabilitation were expected to have polypharmacy, so this study focused on the complexity of prescription. The backgrounds of patients undergoing convalescent rehabilitation, including ADL changes and medication use, were examined. The study participants were predominantly older adults with a mean age of 78.9 years. The “Survey Report on the Current Status and Issues of the Kaifukuki Rehabilitation Ward”published in 2023 reported that the mean age of patients discharged from the Kaifukuki Rehabilitation Ward was 77.3 years, which closely aligns with the mean age of the participants in this study. Therefore, the results of this study have a certain degree of generalizability [19]. The majority of the disease conditions were orthopedic in nature. The mean length of hospital stay was 61.0 days, whereas the above survey report indicated a mean length of hospital stay of 70.5 days [19]. This discrepancy suggests that the relatively shorter length of hospital stay may be partially attributed to the higher proportion of participants with orthopedic diseases in the study population. Those with orthopedic diseases accounted for more than half of the total number of patients in this study. Conceivably, the reported trend of relatively prolonged hospitalization for rehabilitation owing to cerebrovascular diseases and the mean FIM motor [20] and cognitive scores of the study participants improved at the time of discharge compared with those at admission. Furthermore, the MRCI-J scores of all participants for prescription complexity showed an improvement, with a lower mean value at discharge compared with that at admission. Advinha used the MRCI to evaluate the complexity of prescribed drugs in older patients and reported a mean MRCI score of 18.2 [21]. The mean age of the participants in Advinha’s study was 83.9 years [21], which was older than that of the participants in this study; however, the mean MRCI was similar to the results of this study. These findings indicated that the participants in this study had age-appropriate prescription complexities. Interestingly, although pharmacist intervention in the Kaifukuki Rehabilitation Ward is limited owing to the insurance system in Japan, the improvement in the MRCI-J scores of the participants suggests that ward staff recognize the need for medication adjustments and are actively implementing them.

To determine whether the patients’ ADLs affected the MRCI-J scores, the correlation between the FIM motor gains and difference in MRCI-J scores was assessed for all participants, but no correlation was observed. The results suggest that drugs do not uniformly affect ADL changes for patients undergoing convalescent rehabilitation. It was hypothesized that there may be differences in the influence of drugs according to the characteristics of patients’ ADL changes.

Next, we explored whether the participants could be classified according to their transition of ADLs characteristics using hierarchical cluster analysis, as outlined below. In Cluster 1, the mean FIM motor score improved from 51.5 to 80.4 points, reflecting a transition from semi-assistance to independent self-care, whereas the mean FIM cognitive score changed from 29.8 to 31.6 points, which remained stable from the time of admission. In Cluster 2, the mean FIM motor score improved from 28.4 to 68.4 points, reflecting a transition from complete assistance to semi-assistance, whereas the mean FIM cognitive score improved from 25.5 to 29.7 points, which remained stable from the time of admission. In Cluster 3, the mean FIM motor score improved from 73.7 to 87.7 points, further increasing from the original level of independent self-care, whereas the mean FIM cognitive score improved from 34.8 to 35.0 points, maintaining a high level and displaying the highest ADL score among the clusters. Meanwhile, Cluster 4 obtained the lowest ADL score, with a mean FIM motor score improving from 21.6 to 34.2 points. Despite this improvement, the cluster remained in the all-assistance condition. The mean FIM cognitive score also improved from 16.8 to 18.3 points, but the cognitive function remained relatively poor. Suzuki et al. reported falls in patients with stroke, with a median admission FIM motor score (interquartile range [IQR]) of 37 (26–51), median discharge FIM motor score (IQR) of 62 (45–74), median admission FIM cognitive score (IQR) of 20 (13–31), and median discharge FIM cognitive (IQR) of 26 (18–32) for those who experienced multiple falls [13]. The ADL score of Cluster 4 was lower than these results, highlighting the need for increased attention on fall prevention.

Following this, since decision tree analysis based on cluster analysis has been shown to provide more accurate interpretations than traditional decision tree analysis, the same method was used in this study [22]. The characteristics of the eight nodes, classified using the decision tree analysis to identify factors associated with patients stratified by characteristics of ADL transition, are discussed below. Nodes 1 and 2 require long-term treatment. Nodes 1, 2, 5, and 6 were classified according to sex. In the male group of Node 2, most participants were classified as Cluster 4 with limited ADL capacity; in the female group of Node 5, > 80% of the participants were classified as Cluster 3 with high ADL scores. The numbers of participants classified as having nervous system diseases based on the ICD-11 criteria were as follows: 4 of 22 in Node 1, 7 of 20 in Node 2, 0 in Node 5, and 6 of 12 in Node 6, with higher proportions in the male group. Given that neurological diseases are known to affect ADLs, they may have influenced this result. However, as lifestyle and various other factors are also expected to influence the results, careful consideration is needed before attributing the findings solely to sex differences. Nodes 3 and 4 were classified according to the number of rehabilitation units implemented. In Node 3, > 80% of the total participants were classified as Cluster 4, which was associated with limited ADL capacity and an age of > 90 years. In Node 4, participants were similarly elderly, with an age of > 90 years, but were classified as Clusters 1 and 2 and had high ADL scores. Furthermore, rehabilitation was performed more frequently in Node 4 compared with the other nodes, with an average of 3.1 units per day. These results suggest that rehabilitation status may play an important role in the transition of ADLs in older participants > 90 years of age. Forty-one participants were included in Nodes 7 and 8, accounting for 38% of the total study population. Nodes 7 and 8 were classified based on the changes in MRCI-J scores. Node 7 showed stable or improved MRCI-J scores, whereas Node 8 showed worsening MRCI-J scores. In Nodes 7 and 8, > 90% of the participants were not included in Cluster 4 and demonstrated well-maintained ADL capacity upon hospital discharge. Furthermore, the length of hospital stay, age, and rehabilitation status were similar between the groups. Although no correlation with each factor was identified, the fact that the FIM motor gain score of Node 7 was significantly higher than that of Node 8 and was extracted as a relevant factor in the decision tree analysis indicate that changes in prescription in Nodes 7 and 8 had some impact on the ADL status. Elliott et al. stated that drug therapy should not necessarily focus on reducing the number of prescribed medications, but rather on simplifying the complexity of prescriptions when the pharmacist determines that a simpler regimen would benefit the patient [23]. In the recovery phase, rather than a general reduction in the number of prescribed medications, adjustments should be made to support the patient’s rehabilitation, modify or add medications that align with the patient’s lifestyle, or adjust the dosage of medications.

This study has the following limitations. This study’s sample size is small. The goal of this study was to pilot data collection for evidence on the association between the FIM motor gain and difference in MRCI-J scores; in future studies, the survey will be planned to be expanded to include a larger patient population. Moreover, the data were obtained from a single institution and collected retrospectively, and most of the data used in the analysis were treated as continuous variables. The relatively small number of participants in some nodes after the decision tree analysis made comparisons among the nodes challenging. Future research should consider collaborating with other institutions and conducting prospective studies. Additionally, the MRCI-J calculation may not always accurately reflect the actual complexity of prescribing, as factors like crushing tablets may increase the score owing to weighting, even when the actual dose taken by the patient is reduced. In addition, it is difficult to decipher the extent to which the MRCI-J score alone reflects changes in patients’ medication adherence behavior. Consequently, evaluation indices and qualitative research that can reflect the effectiveness of drugs and actual adherence are needed in this regard. Finally, there may be additional confounding factors beyond those examined in this study that could influence the results. Therefore, future studies should account for these potential confounders in their study design.

## Conclusions

No relationship was found between ADLs and prescribed medications in the overall study population. However, in participants comprising Nodes 7 and 8, who had a relatively short length of hospital stay and were discharged with stable physical and cognitive functions, the results suggested that prescription changes had some effect on the ADL status. Therefore, pharmacological interventions may be beneficial in patients classified in Nodes 7 and 8. The results of this study will provide insights on how pharmacological interventions can improve the quality of life of patients in Kaifukuki Rehabilitation Wards.

## Supplementary Information

Supplementary Material 1.

## Data Availability

No datasets were generated or analysed during the current study.

## Abbreviations

ADL: Activities of daily living
FIM: Functional independence measure
HMU: Hyogo Medical University
ICD-11: International Classification of Diseases and Related Health Problems, 11th version
MRCI-J: Japanese version of the medication regimen complexity index
