# Supplementary figures and images for: Identifying characteristics of patients requiring proactive pharmaceutical interventions in the recovery period and assessing the effect of rehabilitation and drugs: a retrospective study
Source: J Pharm Health Care Sci. 2025 Apr 8;11:29. doi: 10.1186/s40780-025-00435-4 (PMC11978153; doi:10.1186/s40780-025-00435-4)

## Slide 1
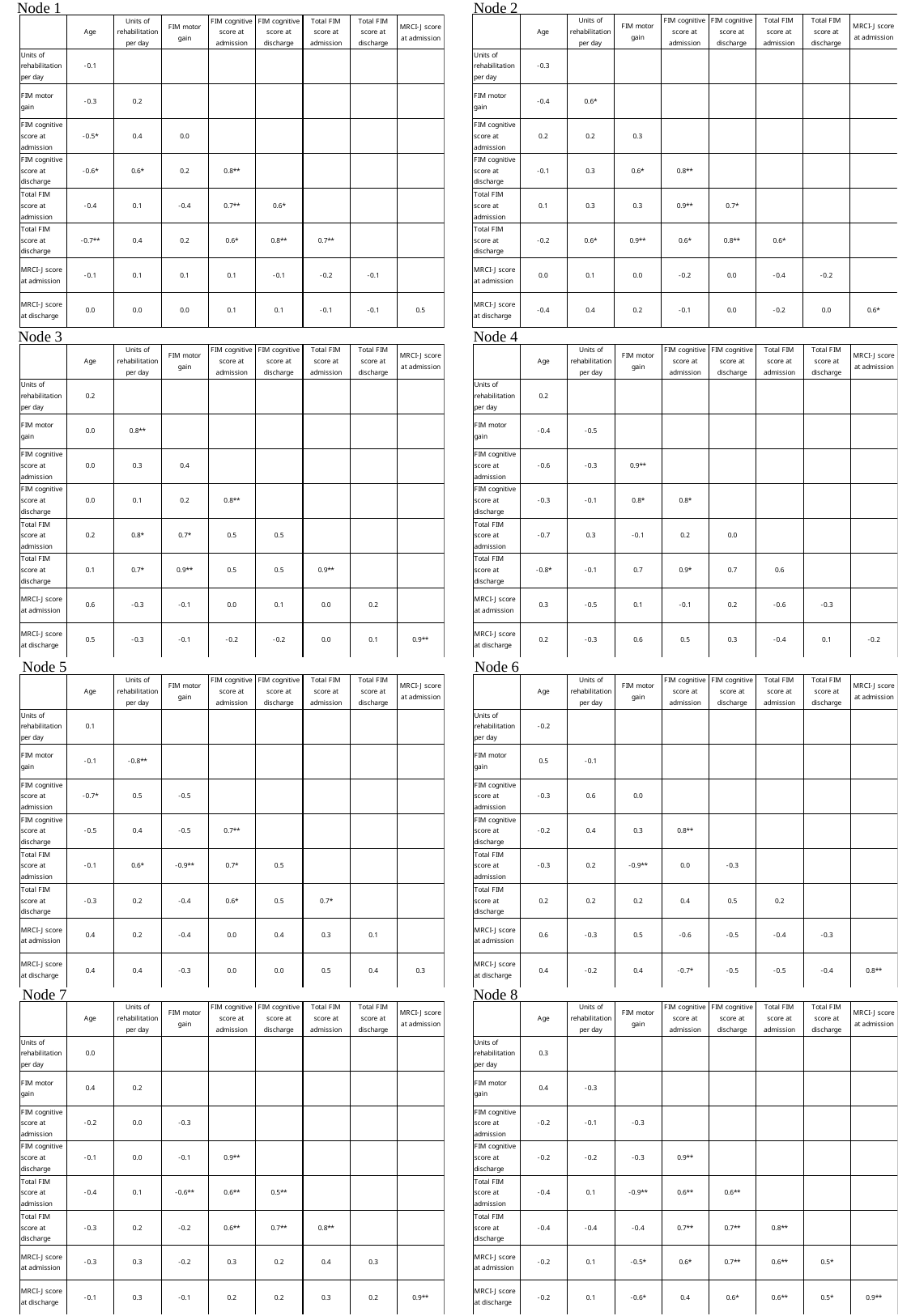

Node 1
Node 2
Node 3
Node 4
Node 5
Node 6
Node 7
Node 8

Supplement: Supplementary file 1 — Supplementary Material 1. [file 40780_2025_435_MOESM1_ESM.pptx]
